# Supplementary material for: Complexome profiling of the Chlamydomonas psb28 mutant reveals TEF5 as an early PSII assembly factor
Source: Plant Cell. 2025 Mar 17;37(6):koaf055. doi: 10.1093/plcell/koaf055 (PMC12164590; doi:10.1093/plcell/koaf055)
Supplement: koaf055_Supplementary_Data [file koaf055_supplementary_data.zip › psb28_interactive.zip!/plots/intensity/qualityPlots/peptides/PSBP6_Cre07.g328200.t1.2.html]

### Cre07.g328200.t1.2

Trivial name: PSBP6  
SeqCoverage [median]: 33.90  
Mass: 33.10 kDa  
  
 

| Sequence | MissedCleavage | Mass [Da] | wtIntensity | muIntensity | Score |
| --- | --- | --- | --- | --- | --- |
| DGQQYWVYEHVSQGSPTITSR | 0 | 2437.13 | 2269.59 | 7915.68 | 63.49 |
| EVALTVLVDR | 0 | 1113.64 | 4123.10 | 14862.04 | 143.03 |
| FALLPTTR | 0 | 917.53 | 11160.13 | 19527.25 | 94.26 |
| IVSEVFDFK | 0 | 1082.56 | 0.00 | 4048.26 | 73.26 |
| NFVTASMTVGPASGVLK | 0 | 1677.88 | 0.00 | 6227.89 | 184.11 |
| PLNMIVSR | 0 | 928.52 | 0.00 | 1834.17 | 91.07 |
| TPEKYSSAAPLTADAR | 1 | 1676.84 | 6246.13 | 4310.88 | 76.34 |
| VALNDVQESHLETR | 0 | 1609.81 | 32303.11 | 45102.52 | 175.29 |
| YSSAAPLTADAR | 0 | 1221.60 | 3234.44 | 12710.02 | 132.88 |
|  |  |  | 59336.5 | 116538.7 |  |
